# HIV antiretroviral exposure in pregnancy induces detrimental placenta vascular changes that are rescued by progesterone supplementation

**DOI:** 10.1038/s41598-018-24680-w

**Published:** 2018-04-26

**Authors:** Hakimeh Mohammadi, Eszter Papp, Lindsay Cahill, Monique Rennie, Nicole Banko, Lakmini Pinnaduwage, Janice Lee, Mark Kibschull, Caroline Dunk, John G. Sled, Lena Serghides

**Affiliations:** 1 Toronto General Hospital Research Institute, University Health Network, 101 College Street, Toronto, Ontario M5G 1L7 Canada; 2 Mouse Imaging Centre, The Hospital for Sick Children, 25 Orde Street, Toronto, Ontario M5T 3H7 Canada; 3 Research Centre for Women’s and Infants’ Health, Lunenfeld Tanenbaum Research Institute, Mount Sinai Hospital, 600 University Avenue, Toronto, Ontario M5G 1X5 Canada; 4 Department of Medical Biophysics, University of Toronto, 101 College Street, Toronto, Ontario M5G 1L7 Canada; 5 Translational Medicine, The Hospital for Sick Children, 686 Bay Street, Toronto, Ontario M5G 0A4 Canada; 6 Department of Immunology and Institute of Medical Sciences, University of Toronto, 1 King’s College Circle, Toronto, Ontario M5S 1A8 Canada; 7 Women’s College Research Institute, Women’s College Hospital, 76 Grenville Street, Toronto, Ontario M5S 1B2 Canada

## Abstract

Adverse birth outcomes are common in HIV-positive pregnant women receiving combination antiretroviral therapy (cART), especially when cART is initiated in early pregnancy. The mechanisms remain poorly understood. Using a mouse model we demonstrate that protease inhibitor based-cART exposure beginning on day 1 of pregnancy was associated with a pro-angiogenic/pro-branching shift in the placenta driven by lower Flt-1 levels and higher Gcm-1 expression. Micro-CT imaging revealed an increase in the number of arterioles in cART-treated placentas, which correlated with fetal growth restriction. Delaying initiation of cART, or supplementing cART-treated mice with progesterone, prevented the pro-angiogenic/pro-branching shift and the associated placenta vascular changes. In agreement with our mouse findings, we observed an increase in the number of terminal-villi capillaries in placentas from HIV-positive cART-exposed women compared to HIV-negative controls. Capillary number was inversely correlated to maternal progesterone levels. Our study provides evidence that cART exposure during pregnancy influences placenta vascular formation that may in turn contribute to fetal growth restriction. Our findings highlight the need for closer investigation of the placenta in HIV-positive pregnancies, particularly for pregnancies exposed to cART from conception, and suggest that progesterone supplementation could be investigated as a possible intervention to improve placenta function in HIV-positive pregnant women.

## Introduction

Combination antiretroviral therapy (cART) has been a resounding success in preventing vertical HIV transmission, with new infections in neonates decreasing by 50% since 2010^1^. However, the use of highly potent drugs during pregnancy comes with potential risks. A growing body of evidence suggests an association between cART use in pregnancy and adverse outcomes including higher rates of stillbirth, pre-term birth (PTB), low birth weight (LBW), and small for gestational age (SGA) births^2–5^. Adverse outcomes may be more pronounced with protease inhibitor based regimens and with exposure to cART prior to conception^2,3,6–9^. Although the benefits of cART use in pregnancy far outweigh the risks, it is important that we improve our mechanistic understanding of the contribution of cART to adverse birth outcomes if we are to optimize treatment for HIV-positive pregnant women and ensure the best maternal and infant outcomes.

The efficient function of the placenta is central to optimal fetal growth^10^. Placenta efficiency is dependent on the appropriate development and remodeling of the uterine and placental vasculatures to meet the metabolic needs of the fetus^11^. Placenta vascular development and remodeling are highly controlled processes mediated primarily by angiogenic factors of the vascular endothelial growth factor (VEGF) and angiopoietin families^12–14^, and regulated by the sex steroid hormones progesterone and estradiol^15–17^. The key pro-angiogenic factors are VEGF and placenta growth factor (PlGF). VEGF interacts with both VEGF receptor 1 (Flt-1) and VEGFR2 (Flk-1), but exerts its pro-angiogenic action primarily via Flk-1, while PlGF interacts with Flt-1^18^. Soluble Flt-1 (sFlt-1) regulates angiogenesis by serving as a decoy receptor for both VEGF and PlGF^19^. Angiopoietin 1 (Ang1) plays a role in stabilizing newly formed vessels, while Ang2 destabilizes vessels to permit vascular remodeling^14^. A balance between pro- and anti-angiogenic factors is important in maintaining placenta vascular plasticity and allowing the placenta to adapt to the changing needs of the fetus. Perturbation in the angiogenic balance has been associated with adverse pregnancy outcomes including pre-eclampsia, fetal growth restriction, and preterm birth^20–23^.

Whether cART use in pregnancy influences angiogenic processes and placenta vascular formation is still an open question. A small number of studies failed to observe an effect of HIV or cART on angiogenic dysregulation in the context of preeclampsia or stillbirth^24,25^. However, HIV protease inhibitors, a class of antiretrovirals that are often included in cART regimens used to treat HIV-positive (HIV+) pregnant women, were shown to decrease VEGF production and to inhibit angiogenesis in cancer cell lines and mouse cancer models, in part by impeding PI3K and Akt activation^26–31^. In a single study using human umbilical vein endothelial cells, treatment with the protease inhibitor indinavir was associated with increased VEGF production and aberrant angiogenesis^28^. Additionally, protease inhibitor-based cART use in pregnancy has been associated with declines in progesterone levels^32,33^. Progesterone plays a key role in directing uterine and placenta angiogenesis, in part by regulating VEGF expression^34–36^.

The objective of this study was to investigate, using a mouse model, whether protease inhibitor-based cART exposure during pregnancy is associated with changes in the levels of angiogenic factors and the morphology of the placental vasculature. We hypothesized that the angiogenic balance required for optimal placenta vascular formation will be altered by cART exposure, contributing to fetal growth restriction.

## Results

### Pregnant mice exposed to cART have low sFlt-1

Pregnant mice were treated from conception until gestational day (GD) 15 (time of sacrifice) with a cART regimen composed of zidovudine/lamivudine/lopinavir/ritonavir (cART) yielding plasma drug levels approximating human minimal effective concentration levels^32^. In agreement with our previous data^32^, we observed smaller fetuses and placentas in the cART-treated mice compared to gestationally matched controls (median [IQR] for fetal weight: cART 0.18 g [0.17–0.20] vs. control 0.23 g [0.20–0.24], p < 0.0001; and for placenta weight: cART 0.076 g [0.071–0.086] vs. control 0.10 g [0.090–0.11], p < 0.0001). Average litter size was smaller and resorptions were more frequent in the cART treated mice (see Supplementary Fig. S1).

**Figure 1:**
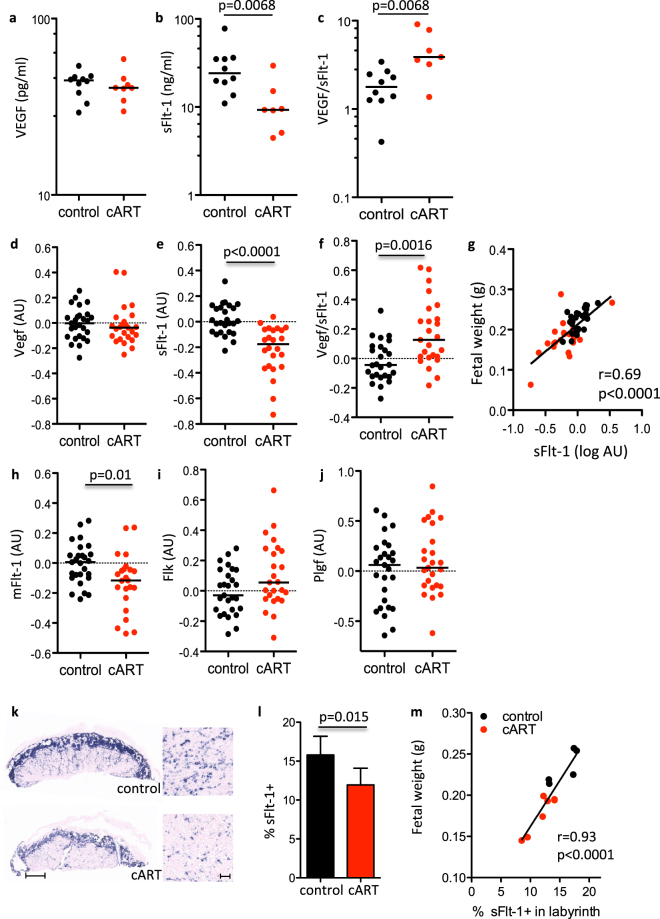
cART exposure during pregnancy is associated with perturbations in the angiogenic balance peripherally and in the placenta. Pregnant mice were treated by gavage with cART (zidovudine/lamivudine/lopinavir/ritonavir) or water as a control starting on GD1 until sacrifice on GD15. Peripheral levels of VEGF (**a**), sFlt-1 (**b**), and the ratio of VEGF to sFlt-1 (**c**) were assessed by EIA in serum collected at GD15 from control (black) and cART-treated (red) pregnant mice. Placental expression levels of Vegf (**d**), sFlt-1 (**e**), mFlt-1 (**h**), Flk (**i**), and Plgf (**j**) were assessed by qPCR in placentas from control (black) and cART-treated (red) mice collected at GD15. The ratio of placenta levels of Vegf to sFlt-1 is shown in (**f**). A correlation between placenta expression levels of sFlt-1 (log transformed) and fetal weight is shown in (**g**). For (**a–c**) n = 10 control dams and n = 8 cART dams. For (**d–j**) n = 26–27 control placentas from 10 dams and n = 23–26 cART placentas from 8 dams. Data are presented as scatter plots with the horizontal line indicating the median. Statistical significance assessed by Mann-Whitney U test. (**k**) Flt-1 expression (stained blue) in control (top) and cART (bottom) placentas assessed by *in situ* hybridization. Full placenta sections are shown on the left (scale bar indicates 1 mm). Sections of the labyrinth are shown on the right (scale bar indicates 200 μm). (**l**) The percentage of Flt-1 expressing cells in the labyrinth zone were estimated by quantifying the area staining blue as a ratio of the total labyrinth area using Image J (version 1.49). n = 5 placentas from 3 dams for control (black) and n = 6 placentas from 4 dams for cART (red). Data presented as mean with SD. Statistical comparison using Student’s t-test. (**m**) Flt-1 expression in the labyrinth assessed by *in situ* hybridization plotted against fetal weight. Correlation assessed by Spearman r.

To assess the angiogenic state, we measured VEGF and sFlt-1 levels in serum from control and cART-treated pregnant mice at GD15. Peripheral levels of the pro-angiogenic factor VEGF were similar between groups, while levels of the anti-angiogenic factor sFlt-1 were significantly lower in cART-treated mice (Fig. 1a–b). cART-treated mice had an elevated VEGF/sFlt-1 ratio (p = 0.0068), indicative of a shift toward a pro-angiogenic state (Fig. 1c).

The placenta serves as the major source of sFlt-1 in pregnancy, so we next examined the mRNA expression levels of Flt-1 and other angiogenic factors in GD15 placentas by qPCR. In agreement with peripheral serum levels, placenta expression levels of Vegf, as well as Plgf, Ang1, Ang2, and Flk-1 were similar between control and cART-treated animals (Fig. 1 and Supplementary Fig. S2). Levels of both the soluble and membrane forms of Flt-1 were lower (p < 0.0001 and p = 0.01 respectively, Fig. 1e,h), and the Vegf to sFlt-1 ratio was higher in placenta from cART-treated mice (Fig. 1f). Expression levels of sFlt-1 directly correlated with fetal weight (r = 0.69, p < 0.0001, Fig. 1g), with placentas having the lowest sFlt-1 expression being associated with the most growth restricted fetuses.

We used *in situ* hybridization to examine the location and expression levels of Flt-1 in the placenta. The percentage of Flt-1 expressing cells was lower in labyrinth zone of the placenta (where oxygen, nutrient, and waste exchange occurs between the mother and fetus) in the cART-treated group compared to control (p = 0.015) (Fig. 1k–l). We also observed a significant correlation between the percentage of Flt-1 expressing cells within the labyrinth zone and fetal weight (r = 0.93, p < 0.0001) (Fig. 1m).

### cART use is associated with an increased number of arterioles in the placenta

To examine the effect of changes in angiogenic factor levels on the placenta vascular network we imaged the feto-placental arterial vasculature at GD15 using micro-computed tomography (micro-CT)^37^. In agreement with a shift toward a pro-angiogenic environment driven by lower sFlt-1 levels, cART-exposed placentas showed increased vascularity compared to controls. The total length of the vascular tree was 1.9x longer, while the average vessel length was 28% shorter in cART-exposed placentas compared to control (see Supplementary Table S1). This pattern suggests increased branching angiogenesis and/or inefficient pruning in cART-exposed placentas, as would be expected with increased VEGF-driven angiogenesis^38,39^.

**Figure 2:**
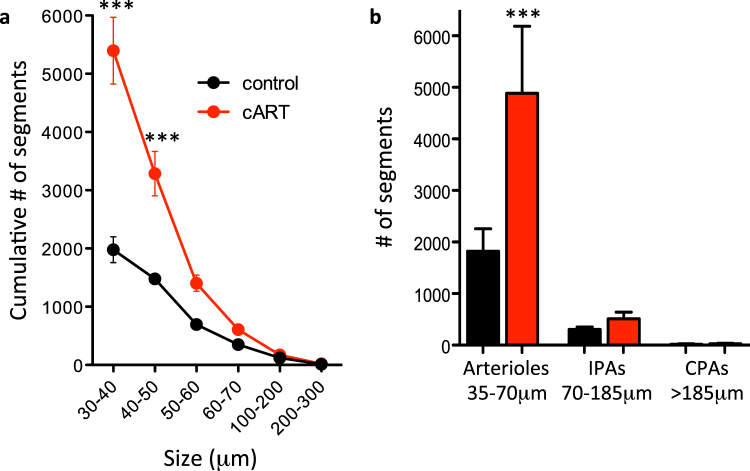
cART exposure during pregnancy is associated with an increase in the number of arterioles in the placenta. Pregnant mice were treated by gavage with cART (zidovudine/lamivudine/lopinavir/ritonavir) or water as a control starting on GD1 until sacrifice on GD15. Placentas were perfused with saline and infused with contrast agent into the arterial vasculature, and scanned using a high-resolution micro-CT scanner. The structure of the vasculature was identified automatically using a segmentation algorithm (see methods for more details). (**a**) Cumulative distribution of vessel diameters in control (black) and cART-treated (red) mice. (**b**) The number of vessel segments within three diameter ranges is shown for control (black) and cART-treated (red) mice. Arterioles (35–70 μm), intraplacental arteries (IPA, 70–185 μm), and chorionic plate arteries (CPA, >185 μm). Data presented as mean with SEM. Statistical comparisons by 2-way ANOVA with Bonferroni post-test in (**a**), and one-way ANOVA with Bonferroni post-test in (**b**). n = 5 placentas from 3 dams for control and n = 6 placentas from 3 dams for cART, ***p < 0.0001.

cART-exposed placentas also had a higher total number of vessel segments compared to controls (Fig. 2a and Supplementary Table S1). The increase in vessel number was confined to vessels of smaller diameter (less than 60 μm) (Fig. 2a). Classifying vessel segments using diameter ranges corresponding to approximate anatomic regions within the vascular tree (i.e. chorionic plate arteries >185 um, intra-placental arteries 70–185 um, and arterioles 35–70 um) demonstrated that the increase in vascularity in cART-exposed placentas was seen primarily in arterioles, while the number of intra-placental and chorionic plate arteries were similar between treatment groups (Fig. 2b). In summary the pro-branching/pro-angiogenic shift observed in cART-exposed mice was reflected in an increase in the number of arterioles in the placenta.

### Gcm-1 expression is upregulated in cART-exposed placentas

**Figure 3:**
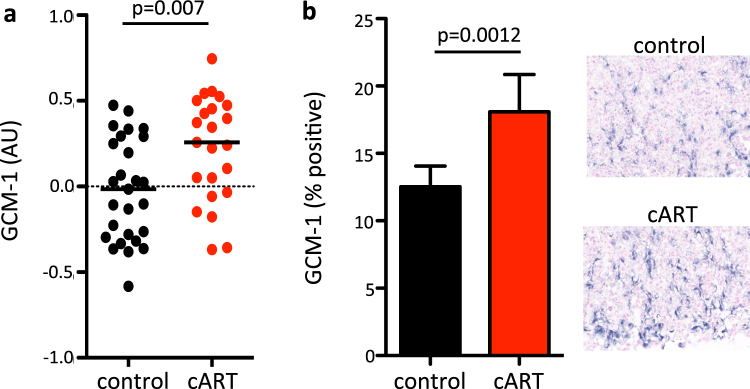
cART exposure during pregnancy is associated with an increase in Gcm-1 expression in the placenta. (**a**) Gcm-1 mRNA expression in the placenta of control (black) and cART-treated (red) mice assessed by qPCR. Data are log transformed and shown as scatter plot with median. Statistical comparison by Mann Whitney U test. n = 27 placentas from 10 dams for control and n = 23 placentas from 8 dams for cART. (**b**) Gcm-1 expressing cells in the labyrinth zone of the placenta of control (black) and cART-treated (red) mice, assessed by *in situ* hybridization. Representative *in situ* images are shown on the right. Control is shown on the top and cART-treated on the bottom. Data are mean with standard deviation. Statistical comparisons using Student’s t-test. n = 6 placentas from 3 dams for control and n = 7 placentas from 4 dams for cART.

One of the key regulators of branching morphogenesis of the villous network that forms the labyrinth in the mouse placenta is Gcm-1^40^. Since villous branching and vascular branching are interconnected processes in the placenta, we examined whether Gcm-1 expression was altered in cART-exposed placentas. Gcm-1 expression was elevated in the placenta of cART-exposed mice compared to controls (p = 0.007, Fig. 3a). *In situ* hybridization experiments also revealed an increase in Gcm-1 positive cells in the labyrinth zone of the cART-exposed placentas (p = 0.0012, Fig. 3b).

### Delayed cART initiation and progesterone supplementation prevent pro-angiogenic shift

**Figure 4:**
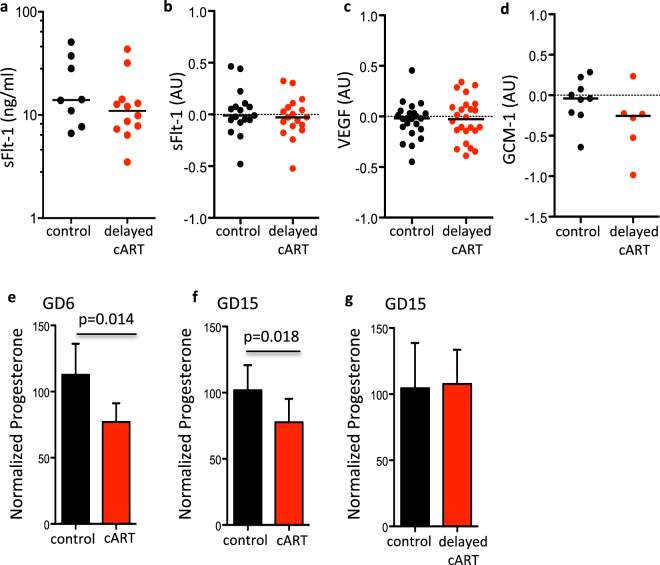
Delaying initiation of cART prevents dysregulation of sFlt-1 and Gcm-1, and progesterone declines. Pregnant mice were treated by gavage with cART (zidovudine/lamivudine/lopinavir/ritonavir) or water as a control starting on GD6 (delayed cART) until sacrifice on GD15. Peripheral levels of sFlt-1 (**a**) were assessed by EIA in serum collected at GD15 from control (black, n = 8) and cART-treated (red, n = 12) pregnant mice. Placental expression levels (log transformed) of sFlt-1 (**b**), Vegf (**c**), and Gcm-1 (**d**) were assessed by qPCR in placentas from control (black) and cART-treated (red) mice collected at GD15. For (**b**) n = 17 placentas from 7 dams for control and n = 18 placentas from 9 dams for cART, for (**c**) n = 23 placentas from 7 dams for control and n = 24 placentas from 9 dams for cART, and for (**d**) n = 9 placentas from 3 dams for control and n = 6 placentas from 3 dams for cART. Statistical comparisons by Mann Whitney U test. Peripheral progesterone levels at GD6 (**e**) or GD15 (**f**) in pregnant mice exposed to control (black), cART starting on GD1 (red), and peripheral progesterone levels at GD15 in pregnant mice exposed to control (black) or cART starting on GD6 (red, delayed cART) (**g**). Data are shown as means with standard deviation. Statistical comparisons by Student’s t-test. For (**e**) and (**f**) n = 10 dams for control and n = 7 dams for cART. For (**g**) n = 7 dams for control and n = 5 dams for delayed cART.

Exposure to protease inhibitor-based cART prior to conception and in the first trimester has been linked to a higher risk for adverse birth outcomes compared to exposure starting in the second trimester^5,8,9,41^. We assessed whether delaying cART treatment of pregnant mice until GD6 (delayed-cART) would prevent the pro-angiogenic shift seen in mice with cART exposure from conception. Serum sFlt-1 levels and placental expression of Vegf, sFlt-1, and Gcm-1 were similar between control and delayed-cART mice (Fig. 4a–d).

Placenta angiogenesis and vascular formation is regulated in part by progesterone^34,35^. We have previously shown that progesterone levels are lower in protease inhibitor-based cART treated HIV-positive pregnant women and in pregnant mice exposed to protease inhibitor-based cART throughout pregnancy^32,33^. We next investigated whether lower progesterone levels could be associated with cART-induced alterations in angiogenic factors. In agreement with our previous observations, progesterone levels were lower in mice exposed to cART throughout pregnancy compared to controls, both at GD6 (p = 0.014) and at GD15 (p = 0.018) (Fig. 4e,f). However, when cART initiation was delayed until GD6, progesterone levels at GD15 were similar to controls (Fig. 4g).

**Figure 5:**
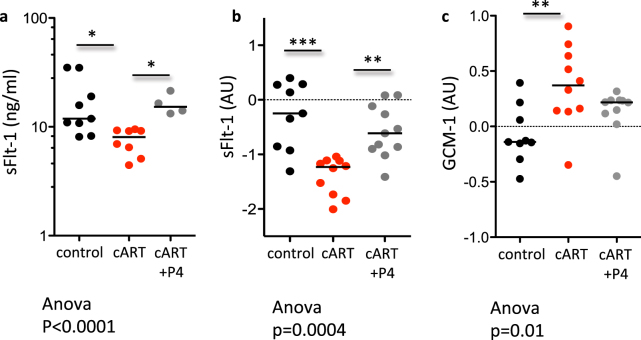
Progesterone supplementation prevents cART-associated dysregulation of sFlt-1 and Gcm-1. Pregnant mice were treated by gavage with cART (zidovudine/lamivudine/lopinavir/ritonavir) or water as a control starting on GD1 until sacrifice on GD15, and were supplemented subcutaneously with either progesterone (0.5 mg in 100 µl of corn oil on GD1, 5, 9, and 13), or corn oil as a control. Peripheral sFlt-1 levels assessed by EIA (**a**), and placental expression levels (log transformed) for sFlt-1 (**b**) and Gcm-1 (**c**) assessed by qPCR are shown for mice treated with control (black), cART (red), or cART plus progesterone (P4) (grey). For (**a**) n = 9 dams for control, n = 8 dams for cART, and n = 4 dams for cART + P4. For (**b**) and (**c**) n = 9 placentas from 5 dams for control, n = 10 placentas from 5 dams for cART, and n = 11 placentas from 5 dams for cART + P4. Statistical comparisons by one way-ANOVA with Bonferroni post-test. *p < 0.05, **p < 0.01, ***p < 0.001.

To test whether cART-associated progesterone declines could directly influence the placenta angiogenic balance, we supplemented mice exposed to cART throughout pregnancy with progesterone and then quantified peripheral levels of sFlt-1, and placental expression of sFlt-1 and Gcm-1. Progesterone supplementation was able to reverse the decline in sFlt-1 levels seen in cART-treated mice in both the placenta and peripherally (Fig. 5a,b). Progesterone supplementation partially reversed the cART-associated increase in Gcm-1 levels in the placenta (Fig. 5c).

### Delayed cART initiation and progesterone supplementation prevents placenta hyper-vascularization

To assess whether the correction in angiogenic factor balance seen with delayed cART initiation and with progesterone supplementation was reflected in the placenta vasculature, we compared the placenta vascular network between control mice, mice exposed to cART throughout pregnancy (cART), mice exposed to cART starting on GD6 (delayed-cART), and mice exposed to cART throughout pregnancy and supplemented with progesterone (cART + P4).

**Figure 6:**
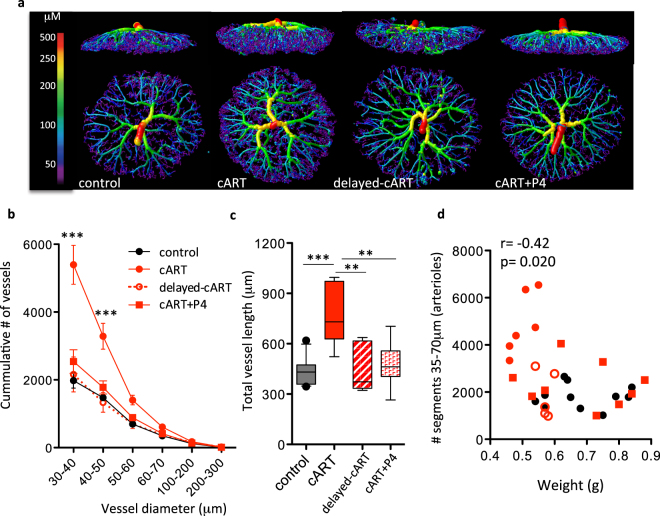
Delaying initiation of cART and supplementing with progesterone prevents the cART-associated placenta vascular changes. (**a**) Representative micro-CT images of GD15 feto-placental arterial vasculature colour coded by vessel diameter from mice treated with control, cART starting on GD1 (cART), cART starting on GD6 (delayed-cART), or cART starting on GD1 and supplemented with progesterone (cART + P4). (**b**) Cumulative number of feto-placental arterial vessels by vessel diameter. Lines represent the mean with SEM. Statistical comparisons by 2-way ANOVA (p < 0.0001) with Bonferroni post-test comparing all treatment groups to control. ***p < 0.001. (**c**) Total vessel length of feto-placental arterial vessels. Box plot shows the median and interquartile range with whiskers denoting the 10^th^–90^th^ percentile, and dots the > 90^th^ percentile. Statistical comparisons by one-way ANOVA (p = 0.0002) with Bonferroni post-test. ***p < 0.001, **p < 0.01. (**d**) Number of arterioles correlate with feto-placenta weight. Data from control mice are shown in black circles, from cART mice in red dots, from delayed-cART mice in open red dots, and from cART + P4 mice in red squares. Correlation assessed by Spearman test. For (**b–d**) n = 11 placentas from 5 dams for control, n = 6 placentas from 3 dams for cART, n = 5 placentas from 2 dams for delayed-cART, and n = 9 placentas from 3 dams for cART + P4.

In contrast to placentas from mice exposed to cART throughout pregnancy, placentas from mice with delayed-cART had vasculatures that were similar to control mice (Fig. 6). Number of vessel segments and total vessel length in the placentas from delayed-cART mice were similar to control and lower than in mice that initiated cART at conception (Fig. 6b,c).

Progesterone supplementation of mice exposed to cART throughout pregnancy also prevented the increase in placenta vascularity. The number of vessel segments and total vessel length in the progesterone-supplemented cART-exposed mice were similar to control and significantly different than cART-exposed unsupplemented mice (Fig. 6).

We observed a negative correlation between the number of arterioles (vessels with diameter between 35 and 70 μm) and fetal unit weight (the weight of the fetus and placenta combined), with placentas with the highest number of arterioles being associated with the smallest fetal unit weights (r = −0.42, p = 0.02) (Fig. 6d). These data suggest that a larger number of small diameter vessels was detrimental to fetal growth.

### The number of terminal villi capillaries is higher in placenta from HIV-positive women on protease inhibitor based-cART

To examine whether our animal model findings are relevant to HIV-positive pregnant women, we counted the number of vessels in immature intermediate and terminal villi in placenta samples collected at delivery from 18 HIV-positive and 13 HIV-negative pregnant women microscopically using stereological principles of random sampling. Demographic and clinical data are shown in Table 1. All women delivered full-term. Nine of the HIV-positive women and none of the HIV-negative delivered an SGA infant. All HIV-positive women were on a protease inhibitor-based cART regimen. Race, body-mass-index (BMI), and mode of delivery were similar between groups, but maternal age was significantly higher in the HIV-positive group (Table 1).

**Table 1:** Demographics and clinical characteristic of human stereology study.

	HIV-negative n = 13	HIV-positive n = 18	p-value
Maternal age (years), median (IQR)	31 (27, 33)	35 (31.5, 36.75)	0.0034
Race, n (%)			0.42
Black	9 (69%)	15 (83%)	
White	3 (23%)	3 (17%)	
Other	1 (8%)	0 (0%)	
BMI, mean (SD)	26.4 (6.0)	26.5 (5.0)	0.95
Gestational length (weeks), median (IQR)	39.57 (38.9, 40)	39.3 (38.4, 40.1)	0.56
Small for gestational age, n (%)	0 (0%)	9 (50%)	0.002
Placenta weight (g), median (IQR)	534 (450, 585)	450 (354, 513)	0.037
Delivery mode, n (%)			0.93
Vaginal	7 (54%)	10 (56%)	
c-section	6 (46%)	8 (44%)	
CD4 count, median (IQR)	N/A	439 (360, 589)	
Viral load, median (IQR)	N/A	40 (40, 40)	
Detectable viral load at recruitment, n (%)	N/A	4 (22%)	
Viral load above 1000, n (%)	N/A	1 (6%)	
Capillary count, mean (SD)	3.60 (0.35)	4.45 (1.08)	0.0039
Maternal progesterone levels at delivery* mean (SD) (log ng/ml)	2.41 (0.31)	2.15 (0.27)	0.08

^*^n = 9 for HIV-negative and n = 12 for HIV-positive. IQR, interquartile range; SD, standard deviation; N/A, not applicable; BMI, body mass index.

**Figure 7:**
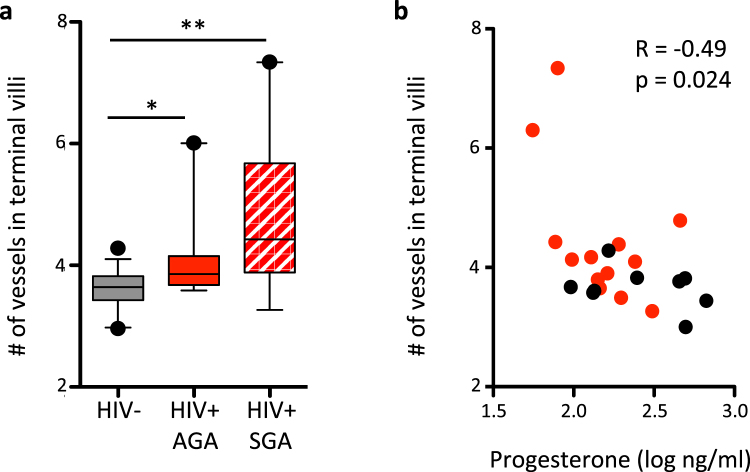
The number of terminal villi capillaries is higher in placenta from HIV-positive women on PI-cART. (**a**) The number of capillaries in immature intermediate and terminal villi was assessed in term placenta sections from HIV-negative (HIV−, grey), HIV-positive women on cART with an average for gestational age (HIV+ AGA) birth (solid red), and HIV-positive women on cART with a small for gestational age (HIV+ SGA) birth (stripped red). Data shown as box (showing median and interquartile range) and whisker (showing 10^th^ to 90^th^ centile) plots. Dots signify values outside the 10^th^ and 90^th^ percentile. Statistical comparison by Kruskal Wallis test (p = 0.0076) with Dunn’s post-test, n = 13 for HIV-negative and n = 9 for HIV-positive AGA and n = 9 for HIV-positive SGA. *p < 0.05, **p < 0.01. (**b**) Correlation between capillary number and progesterone levels in maternal blood at delivery. Correlation assessed by Pearson R test.

The number of vessels in the immature intermediate and terminal villi was higher in the HIV-positive group (mean [SD]; HIV-positive: 4.45 [1.08] vs. HIV-negative: 3.60 [0.35], p = 0.0039). When we stratified the data by SGA status, we observed a stepwise increase in number of vessels with the lowest number of vessels seen in the HIV-negative group and the higher number in the HIV-positive SGA group (Fig. 7). We also observed a negative correlation between vessel number and progesterone levels in the maternal blood at delivery (R = −0.49, p = 0.024). The association between vessel number and maternal progesterone levels remained significant after correcting for SGA status and maternal age (coefficient [95% confidence interval] unadjusted: −1.57 [−2.83, −0.31], p = 0.014; adjusted: −1.42 [−2.70, −0.14], p = 0.03).

## Discussion

Our data demonstrate an association between cART exposure in pregnancy and placenta vascular changes. Mice exposed to cART throughout pregnancy showed evidence of a perturbed angiogenic balance with a shift towards a more pro-angiogenic/pro-branching state driven by lower levels of both soluble and membrane Flt-1. This pro-angiogenic/pro-branching state was reflected in the placenta vasculature, with placentas from cART-treated mice having a higher number of small diameter vessels (arterioles) compared to controls, consistent with an increase in branching angiogenesis. In agreement with these findings we also observed higher levels of Gcm-1 in the placenta of cART-treated mice, a transcription factor known to regulate placenta branching morphogenesis^42^. Both lower sFlt-1 levels and higher number of arterioles were associated with lower fetal weights. Delaying the initiation of cART until after GD6, or supplementing cART-treated mice with progesterone, prevented the pro-angiogenic shift and the increase in number of arterioles in the placenta. Extending our animal model findings to a human population, we observed a higher number of vessels in the immature intermediate and terminal villi of placentas from HIV-positive women on protease inhibitor based-cART compared to HIV-negative women. In addition, we observed an indirect correlation between vessel number and maternal progesterone levels at delivery.

Placenta angiogenesis and vascular formation is regulated in part by progesterone^34,35^. We have previously shown that progesterone levels are lower in protease inhibitor-based cART treated HIV-positive pregnant women and in pregnant mice exposed to protease inhibitor-based cART throughout pregnancy^32,33^. In agreement with our previous observations, progesterone levels (both in early and late gestation) were lower in mice exposed to cART throughout pregnancy compared to controls. However, when cART initiation was delayed until GD6, progesterone levels did not decline. To test whether cART-associated progesterone declines could directly influence the placenta angiogenic balance, we supplemented mice exposed to cART throughout pregnancy with progesterone and then assessed peripheral levels of sFlt-1, and placental expression of sFlt-1 and Gcm-1. Progesterone supplementation was able to reverse the decline in sFlt-1 levels seen in cART-treated mice in both the placenta and peripherally. Progesterone supplementation also partially reversed the cART-associated increase in Gcm-1 levels in the placenta.

While anti-angiogenic properties have been reported for cART and protease inhibitors in cancer models^30,43,44^, we observed a shift towards a detrimental pro-angiogenic placental state in the context of cART use in pregnancy. We hypothesize that this imbalance is a compensatory mechanism for a cART-associated insult early in pregnancy likely linked to progesterone dysregulation, as delaying cART initiation until after GD6 prevented the pro-angiogenic shift and placenta vascular changes. The first 6 days of a mouse pregnancy are dominated by the decidualization of the uterine endometrium to form the decidua (the maternal side of the placenta)^45^. Progesterone is a critical regulator of endometrial decidualization and uterine angiogenic factor expression, and plays a role in uterine vessel remodeling in early pregnancy (a process that ensures adequate maternal perfusion of the placenta)^34,35,46^. The lower levels of progesterone we observed at GD6 in the cART-exposed mice may contribute to a defect in these important early pregnancy processes that could lead to increased uterine artery resistance and poor maternal vascular perfusion, which may drive a compensatory pro-angiogenic state in the placenta. This hypothesis is supported by our findings that progesterone supplementation of cART-treated mice corrects the angiogenic balance and prevents the hyper-vascularization of the placenta. And although small in number, our human data also support an association between lower progesterone levels and placenta hyper-vascularization. Of note, maternal vascular malperfusion (a condition strongly associated with hypertensive disorders of pregnancy) was 2.87 times more likely in HIV-positive pregnant women (after adjusting for smoking, CD4 count, and parity), although these women were not hypertensive^47^. An association with cART was observed although it did not reach significance^47^. Future studies will need to address the impact of cART on uterine decidualization and vessel remodeling, and examine the downstream effects on placenta function and efficiency. This is especially relevant given current treatment guidelines that promote early cART initiation^48^, and increasing data that suggest higher rates of adverse birth outcomes for infants exposed to cART from conception^5,8,9,41^. Progesterone supplementation of HIV-positive pregnant women may also merit further investigation as a means of improving placenta efficiency and fetal growth^49^.

A limited number of studies have investigated sFlt-1 levels in HIV-positive pregnant women. A South African study compared sFlt-1 levels in preeclamptic and normotensive HIV-positive and HIV-negative pregnant women. Although sFlt-1 levels were higher in preeclamptic women irrespective of HIV status, a trend toward lower serum sFlt-1 levels was seen in the HIV-positive compared to HIV-negative women^25^. All HIV-positive women were on cART but details on the regimens were not available. In a separate study, sFlt-1 levels were measured in cART-naive HIV-positive women at enrolment (26–34 weeks gestation) and 1-month post-cART initiation^24^. As with the previous study, sFlt-1 levels increased in women who developed preeclampsia. However in the non-preeclamptic women a trend toward a decline in sFlt-1 levels at 1-month post cART-initiation was observed^24^, although sFlt-1 levels are expected to increase during that time in gestation^50^. These data and our findings highlight the need for further study of angiogenic dysregulation and placenta function in HIV-positive cART-exposed pregnant women.

Although our studies do not directly implicate Flt-1 as the mediator of the placenta vascular changes, lower Flt-1 levels likely contributed to the higher number of arterioles in the placentas of cART-treated mice. These data are in agreement with previous observations of endothelial overgrowth and increased vascularity in mice deficient in Flt-1^51,52^. The increase in the number of arterioles in cART-exposed placentas may be the result of increased branching angiogenesis due to lower sFlt-1 and mFlt-1 levels. sFlt-1 is a potent decoy receptor for VEGF, limiting its availability to signal through Flk-1. Lower sFlt-1 levels would result in more VEGF available to interact with Flk-1 and stimulate placental vessel branching^53,54^. In addition, excess free VEGF and lower mFlt-1 levels may interfere with PlGF signaling, limiting non-branching angiogenesis^18^, and the development of the capillary system of the mouse placental labyrinth.

The higher levels of Gcm-1 may also contribute to increased vessel branching. Gcm-1 is specifically expressed in the placenta of both mice and humans, and is involved in placenta branching morphogenesis, a process that occurs simultaneously with the development of the placenta vascular network^55^. In Gcm-1 deficient mice there is a complete lack of branching at the chorioallantoic interface resulting in embryonic lethality^42^. Although it seems plausible that higher Gcm-1 expression should correlate with increased placental vessel branching as seen in our study, the interrelation between villous and vessel branching and the role Gcm-1 plays may be more complicated, as a study of Gcm-1 heterozygous placentas demonstrated increased branching of the feto-placental arterial tree in comparison to Gcm-1 wild-type placentas^56^.

The placenta vasculature changes seen in our cART-treated mice and in our HIV-positive cART-exposed women were associated with fetal growth restriction in mice and SGA births in the women, suggesting that the increase in the number of arteriole-sized vessels may be associated with suboptimal placenta function. This type of vascular pattern (increased number of small diameter vessels) has been associated with increased calculated vascular resistance in both eNOS−/− mice and in malaria infected mice^57,58^. Increased vascular impedance in the feto-placenta vasculature could lead to inefficient nutrient and oxygen transport to the fetus contributing to poor fetal growth.

Intrauterine growth restriction (IUGR) is thought to stem from a hypo-vascular feto-placental tree with decreased numbers of mature intermediate and terminal villi^59,60^. Although cART-exposed murine fetuses were growth restricted, the feto-placenta vascular pattern of cART-exposed placentas was hyper-vascular with increased vessel branching– opposite to what has been reported for IUGR – however, both configuration would be expected to lead to increased feto-placental vascular resistance^59^. A similar increase in vessel number was observed in the terminal villi of HIV-positive cART exposed placentas particularly those associated with an SGA birth. While our findings will need to be confirmed in a larger clinical study, our data suggest that placenta vascular changes leading to fetal growth restriction in the context of cART may be linked to a failure in the switch between branching and non-branching angiogenesis necessary for the development of the high-flow low-resistance feto-placental circulation, and may differ from placenta vascular changes of classically defined IUGR^61^.

Our study has several limitations. Our animal model is limited to the study of cART and does not include HIV. HIV infection is also associated with alterations in angiogenic factors including VEGF, inflammation, immune dysregulation, and complement activation^62^, all of which could influence placenta angiogenesis and vascular formation, and modify any cART effects. Additionally, our animal studies are limited to a single cART regimen. We are not able to distinguish whether specific components of the regimen contributed to vascular changes, or if other cART regimens would result in similar angiogenic dysregulation and alteration in the placental vasculature. Although we present supporting data showing an increase in vessel number in immature intermediate and terminal villi in placenta from HIV-positive cART-exposed pregnant women compared to controls, the small number of participants included in our study is a limitation. In addition, our human studies were limited to comparisons between HIV-negative and HIV-positive women on cART making it impossible to delineate the effects of HIV from cART on the placenta vasculature. Given treatment guidelines, inclusion of an HIV-positive untreated comparator arm would be unethical. Additionally, because all HIV-positive women included in this study received a protease inhibitor-based regimen (due to treatment recommendations at time of recruitment) we were unable to determine whether effects on the placenta vasculature were specifically due to protease inhibitor exposure. Future studies including women on a variety of cART regimens are merited.

In conclusion, using a mouse pregnancy model, we present evidence that exposure to cART from conception is associated with angiogenic dysregulation and excessive small diameter vessel branching in the feto-placental arterial tree that correlate with fetal growth restriction. Delaying cART initiation or supplementing cART-treated mice with progesterone rescued the phenotype. We further present supporting evidence from a small number of pregnant women, showing a higher number of vessels in immature intermediate and terminal villi of placentas from HIV-positive compared to HIV-negative pregnant women, and an inverse correlation between progesterone levels and vessel number. Our findings highlight the need for closer investigation of placental structural and functional changes in HIV-positive pregnant women, particularly those with cART exposure from conception. Our data also suggest that progesterone supplementation could be investigated as a possible intervention to improve placenta efficiency in HIV-positive pregnant women on cART.

## Materials and Methods

### Mouse model

Animal experiments were approved by the University Health Network Animal Use Committee and performed according to the policies and guidelines of the Canadian Council on Animal Care. Housing conditions, breeding, and drug treatment of mice have been described previously^32^. Plugged C57Bl/6 females were randomly assigned into a treatment arm, and administered either zidovudin/lamivudine (100/50 mg/kg/day) plus lopinavir/ritonavir (33/8.3 mg/kg/day), or water as a control by oral gavage once daily starting on day of plug detection (GD1) until sacrifice. In progesterone supplementation experiments, 0.5 mg of progesterone in 100 µl of corn oil was administered subcutaneously on GD1, 5, 9, and 13. Control mice received 100 µl of corn oil. In delayed-cART experiments, mice were administered cART or water as control (as above) once daily starting on GD6 until sacrifice. Animals were euthanized by CO_2_ inhalation, or by cervical dislocation if they were to be used for micro-CT studies. Blood was collected by cardiac puncture. Fetal and placenta weight were recorded. Placentas were snap frozen for RNA extraction or fixed in 10% formalin for histological and *in-situ* examination. Details on placenta collection for micro-CT studies are shown below.

### Biochemical parameters

Mouse sFlt-1 and VEGF (R&D Systems) were quantified in serum using commercially available enzyme immunoassay (EIA) kits according to manufacturers’ instructions. Human and mouse progesterone was quantified using a competitive EIA according to the manufacturer’s instructions (DRG International and Cayman Chemical). Samples were assayed in duplicate.

### RNA extraction and real-time quantitative polymerase chain reaction (qPCR)

RNA was extracted from placental tissue using the Mirvana RNA extraction kit (Life Technologies). RNA quantity and purity were assessed using NanoDrop 2000c (Thermo Scientific). Only samples with a 260 nm to 280 nm absorption ratio of >2.0 for RNA were used. cDNA was produced from 50 ng of RNA using Invitrogen reverse transcriptase kit. cDNA (1ng) was amplified in triplicated with SYBR Green I Master mix (Roche) and forward and reverse primers (see Supplementary Table S2) in a LightCycler480 instrument (Roche). Multiple housekeeping genes were tested (β-actin, 18S rRNA, YWHAZ, HPRT1, and the reproductive-organ specific housekeeping gene Rpl-19). The most robust and least variable housekeeping gene was HPRT1, and was selected as the reference gene. The relative quantification (ΔΔCT) method was used for gene-expression calculations^63^.

### *In-situ* hybridization

*In situ* hybridization was performed on 7 μm cross-sections of formalin fixed and paraffin embedded placental tissues, using digoxygenin-labelled RNA probes for Gcm-1 and Flt1. Processing of specimens, probe labeling and colorimetric detection was performed as previously described^64^. Plasmid pBluescripts-KSII-Flt1 comprising exons 1–10 of mouse Flt-1 mRNA^65^ was digested with NotI and transcribed with T3 enzyme to generate an antisense probe; for sense probe a PstI digest and T7 transcription enzyme was used. Gcm-1 antisense (XhoI digest, T7 transcription) and sense (XbaI digest, T3 transcription) probes were generated from plasmid pBluescript-KSII-Gcm-1 comprising the complete murine open reading frame^66^. Placenta sections were digitally scanned and the percentage of Flt-1 expressing cells in the labyrinth zone were estimated by quantifying the area staining blue as a ratio of the total labyrinth area using Image J (version 1.49).

### Micro-CT

Detailed methods for preparing the fetoplacental vasculature for micro-CT imaging have been described previously^67,68^. Briefly, on GD15 pregnant dams were sacrificed by cervical dislocation. Uteri were removed and immersed in ice-cold phosphate buffered saline (PBS). Each fetal unit was extracted from the uterus while maintaining the vascular connection of the fetus to the placenta, and bathed in warm PBS to resume blood circulation. The fetal unit was weighed. The umbilical artery was cannulated and the placenta was perfused with saline (with heparin, 100 units/mL) to clear blood from the vasculature. Radio-opaque silicone rubber contrast agent (Microfil; Flow Technology) was infused into the arterial vasculature. After the agent polymerized, the placenta was separated and fixed (10% formalin) for 24–48 hours at 4 °C. Specimens were mounted in 1% agar with 10% formalin and scanned using a Bruker SkyScan1172 high-resolution micro-CT scanner at a resolution of 7.1 μm. 996 views were acquitted via 180-degree rotation with an X-ray source at 54 kVp and 185 µA. Three-dimensional micro-CT data were reconstructed using SkyScan NRecon software. The structure of the vasculature was identified automatically using a segmentation algorithm as described in detail previously^37^. The leaves of the vascular tree were pruned to 35 μm to improve data consistency. Analysis was performed on control (n = 11), cART (n = 6), delayed-cART (n = 5), and cART + P4 (n = 9) placentas. Each group contains a minimum of 2 dams and 1–3 specimens per litter.

### Human samples

The ethics boards of University Health Network, St. Michael’s Hospital, Mount Sinai Hospital, and Women’s College Hospital in Toronto, Canada approved this study. All experiments were performed in accordance with the Tri-Council Policy Statement on Ethical Conduct for Research Involving Humans (TCPS). Written informed consent was collected from all participants. Placenta samples and maternal heparinized plasma samples collected at delivery were obtained from women who consented to participate in a biobank program for the purpose of facilitating research in HIV and pregnancy. Details on the inclusion and exclusion criteria have been described^32^. Samples from 18 HIV-positive (9 with average for gestational age births and 9 with small for gestational age births) and 13 HIV-negative participants were randomly selected from women that delivered full-term. All HIV-positive participants received a PI-based cART (8 on lopinavir/rit, 7 on atazanavir/rit, and 3 on darunavir/rit based regimen). Thirteen of the 18 HIV-positive women came into the pregnancy already on cART, 5 initiated cART in the first trimester, and 1 initiated cART in the second trimester. Demographic and clinical characteristics are shown in Table 1.

### Placenta image analysis

Stereological principles of random sampling were utilized in the collection of placenta biopsies and generation of histological slides. Full thickness-core placenta sections (3 per placenta) were fixed in formalin and paraffin embedded. 5 µm sections at random angles were sectioned from each block. Three random sections from each placenta block were stained with Masson’s Trichrome stain, and evaluated microscopically using an Olympus BX61 upright, motorized microscope with Olympus DP72 digital colour camera (Olympus Canada). Capillary number per villous was counted in immature intermediate and terminal villi using newCAST software (Visopharm). Firstly, an area mask was set on the peripheral placenta excluding the large mature placenta stem villi. Visual fields from 3% of each section were randomly selected by the software and analysed at 10x magnification. Capillaries within villi cross-sections lying within the software provided box-probes were counted. All assessments of capillary count were performed blinded to HIV status and pregnancy outcome.

### Statistical methods

For continuous variables, means/medians were compared using Student’s t-test or Mann-Whitney U test as appropriate. Categorical variable were compared using Chi squared or Fisher’s exact test. For all mRNA expression experiments, qPCR data was log-transformed prior to statistical analyses. For comparisons of more than two groups, one-way ANOVA with Bonferroni post-test or Kruskal-Wallis test with Dunn’s post-test was used as appropriate. For analysis of vessel number across vessel size and treatment two-way ANOVA with Bonferroni post-test was used. Correlation was assessed by Spearman rank or Pearson R correlation coefficient. Generalized linear models were used to examine the relationship between capillary number and maternal progesterone levels, SGA, and maternal age. A two-sided p-value of less than 0.05 was used as the cut-off for statistical significance. All statistical analyses were performed using STATA (v12.0) and GraphPad Prism (v5.0).

## Electronic supplementary material

Supplemental figures and tables

## References

[1] UNAIDS. Global AIDS Update 2016.

[2] Chen JY (2012). Highly active antiretroviral therapy and adverse birth outcomes among HIV-infected women in Botswana. J. Infect. Dis..

[3] Li N (2016). Antiretroviral therapy in relation to birth outcomes among HIV-infected women: a cohort study. J. Infect Dis..

[4] Mofenson, L. M. Antiretroviral therapy and adverse pregnancy outcome: the elephant in the room? *J. Infect. Dis*. **213**, 1051–1054. 10.1093/infdis/jiv390 26265779

[5] Fowler MG (2016). Benefits and risks of antiretroviral therapy for perinatal HIV prevention. N. Engl. J. Med..

[6] Zash R (2017). Comparative safety of antiretroviral treatment regimens in pregnancy. JAMA Pediatr..

[7] Kourtis AP, Schmid CH, Jamieson DJ, Lau J (2007). Use of antiretroviral therapy in pregnant HIV-infected women and the risk of premature delivery: a meta-analysis. AIDS..

[8] Sibiude J (2012). Premature delivery in HIV-infected women starting protease inhibitor therapy during pregnancy: role of the ritonavir boost?. Clin. Infect. Dis..

[9] Van Dyke RB, Chadwick EG, Hazra R, Williams PL, Seage GR (2016). The PHACS SMARTT study: assessment of the safety of in utero exposure to antiretroviral drugs. Front. Immunol..

[10] Kingdom J, Huppertz B, Seaward G, Kaufmann P (2000). Development of the placental villous tree and its consequences for fetal growth. Eur. J. Obstet. Gynecol. Reprod. Biol..

[11] Burton GJ, Fowden AL, Thornburg KL (2016). Placenta origins of chronic disease. Physiol. Rev..

[12] Redmer DA, Aitken RP, Milne JS, Reynolds LP, Wallace JM (2005). Influence of maternal nutrition on messenger RNA expression of placental angiogenic factors and their receptors at midgestation in adolescent sheep. Biol. Reprod..

[13] Luther J (2007). Placental growth, angiogenic gene expression, and vascular development in undernourished adolescent sheep. Biol. Reprod..

[14] Geva E (2002). Human placental vascular development: vasculogenic and angiogenic (branching and nonbranching) transformation is regulated by vascular endothelial growth factor-A, angiopoietin-1, and angiopoietin-2. J. Clin. Endocrinol. Met..

[15] Albrecht ED, Pepe GJ (2010). Estrogen regulation of placental angiogenesis and fetal ovarian development during primate pregnancy. Int. J. Dev. Biol..

[16] Spencer TE, Bazer FW (2002). Biology of progesterone action during pregnancy recognition and maintenance of pregnancy. Front. Biosci..

[17] Chen JZ, Sheehan PM, Brennecke SP, Keogh RJ (2012). Vessel remodelling, pregnancy hormones and extravillous trophoblast function. Mol. Cell Endocrinol..

[18] Ahmed A, Dunk C, Ahmad S, Khaliq A (2000). Regulation of placenta vascular endothelial growth factor (VEGF) and placenta growth factor (PlGF) and soluble Flt-1 by oxygen—a review. Placenta..

[19] Zygmunt T (2011). Semaphorin-PlexinD1 signaling limits angiogenic potential via the VEGF decoy receptor sFlt1. Dev. Cell..

[20] Stepan H, Unversucht A, Wessel N, Faber R (2007). Predictive value of maternal angiogenic factors in second trimester pregnancies with abnormal uterine perfusion. Hypertension..

[21] Espinoza J (2007). Identification of patients at risk for early onset and/or severe preeclampsia with the use of uterine artery Doppler velocimetry and placenta growth factor. Am. J. Obstet. Gynecol..

[22] Romero R (2008). A longitudinal study of angiogenic (placenta growth factor) and anti-angiogenic (soluble endoglin and soluble vascular endothelial growth factor receptor-1) factors in normal pregnancy and patients destined to develop preeclampsia and deliver a small for gestational age neonate. J. Matern. Fetal Neonatal. Med..

[23] Maynard SE (2003). Excess placental soluble fms-like tyrosine kinase 1 (sFlt1) may contribute to endothelial dysfunction, hypertension, and proteinuria in preeclampsia. J. Clin. Invest..

[24] Powis KM (2013). High viral load and elevated angiogenic markers associated with increased risk of preeclampsia among women initiating highly active antiretroviral therapy in pregnancy in the Mma Bana study, Botswana. J. Acquir. Immune Defic. Syndr..

[25] Govender N, Naicker T, Rajakumar A, Moodley J (2013). Soluble fms-like tyrosine kinase-1 and soluble endoglin in HIV-associated preeclampsia. Eur. J. Obstet. Gynecol. Reprod. Biol..

[26] Esposito V (2006). Evaluation of antitumoral properties of the protease inhibitor indinavir in a murine model of hepatocarcinoma. Clin. Cancer Res..

[27] Pore N, Gupta AK, Cerniglia GJ, Maity A (2006). HIV protease inhibitors decrease VEGF/HIF-1 alpha expression and angiogenesis in glioblastoma cells. Neoplasia.

[28] Di Simone N (2007). Effects of antiretroviral therapy on tube-like network formation of human endothelial cells. Biol. Pharm. Bull..

[29] Chow WA, Jiang C, Guan M (2009). Anti-HIV drugs for cancer therapeutics: back to the future?. Lancet Oncol..

[30] Sgadari C (2002). HIV protease inhibitors are potent anti-angiogenic molecules and promote regression of Kaposis sarcoma. Nat. Med..

[31] Toschi E (2011). Human immunodeficiency virus protease inhibitors reduce the growth of human tumors via a proteasome-independent block of angiogenesis and matrix metalloproteinases. Int. J. Cancer..

[32] Papp E (2015). HIV protease inhibitor use during pregnancy is associated with decreased progesterone levels, suggesting a potential mechanism contributing to fetal growth restriction. J. Infect. Dis..

[33] Papp E (2016). Low prolactin and high 20-α-hydroxysteroid dehydrogenase levels contribute to lower progesterone levels in HIV-infected pregnant women exposed to protease inhibitor-based combination antiretroviral therapy. J. Infect. Dis..

[34] Kim M (2013). VEGF-A regulated by progesterone governs uterine angiogenesis and vascular remodeling during pregnancy. EMBO Mol. Med..

[35] Chen JZ, Wong MH, Brennecke SP, Keogh RJ (2011). The effects of human chorionic gonadotrophin, progesterone and oestradiol on trophoblast function. Mol. Cell. Endocrinol..

[36] Walter LM, Rogers PA, Girling JE (2005). The role of progesterone in endometrial angiogenesis in pregnant and ovariectomised mice. Reproduction..

[37] Rennie MY (2011). Vessel tortuousity and reduced vascularization in the fetoplacental arterial tree after maternal exposure to polycyclic aromatic hydrocarbons. Am. J. Physiol. Heart. Circ. Physiol..

[38] Bussolati B (2001). Vascular endothelial growth factor receptor-1 modulates vascular endothelial growth factor-mediated angiogenesis via nitric oxide. Am. J. Pathol..

[39] Gerhardt H (2003). VEGF guides angiogenic sprouting utilizing endothelial tip cell filopodia. J. Cell Biol..

[40] Cross JC, Nakano H, Natale DR, Simmons DG, Watson ED (2006). Branching morphogenesis during development of placenta villi. Differentiation..

[41] Watts DH (2013). Combination antiretroviral use and preterm birth. J. Infect. Dis..

[42] Anson-Cartwright L (2000). The glial cells missing-1 protein is essential for branching morphogenesis in the chorioallantoic placenta. Nat. Genet..

[43] Pati S (2002). Antitumorigenic effects of HIV protease inhibitor ritonavir: inhibition of Kaposi sarcoma. Blood..

[44] Maggiorella L (2005). Combined radiation sensitizing and anti-angiogenic effects of ionizing radiation and the protease inhibitor ritonavir in a head and neck carcinoma model. Anticancer Res..

[45] Croy, B. A., Yamada, A. T., DeMayo, F. J. & Adamson, S. L. The guide to investigation of mouse pregnancy. (AcademicPress, UK, ed. 1, 2014).

[46] Kaya HS (2015). Roles of progesterone receptor A and B isoforms during human endometrial decidualization. Mol. Endocrinol..

[47] Kalk E (2017). Placenta pathology in HIV infection at term: a comparison with HIV-uninfected women. Trop. Med. Int. Health..

[48] Lifson AR (2017). Improved quality of life with immediate versus deferred initiation of antiretroviral therapy in early asymptomatic HIV infection. AIDS..

[49] Siou K (2016). Progesterone supplementation for HIV-positive pregnant women on protease inhibitor-based antiretroviral regimens (the ProSPAR study): a study protocol for a pilot randomized controlled trial. Pilot Feasibility Stud..

[50] Noori M, Donald AE, Angelakopoulou A, Hingorani AD, Williams DJ (2010). Prospective study of placental angiogenic factors and maternal vascular function before and after preeclampsia and gestational hypertension. Circulation..

[51] Fong GH, Rossant J, Gertsenstein M, Breitman ML (1995). Role of the Flt-1 receptor kinase in regulating the assembly of vascular endothelium. Nature..

[52] Jin J (2012). Soluble FLT1 binds lipid microdomains in podocytes to control cell morphology and glomerular barrier function. Cell..

[53] Wang A, Rana S, Karumanchi SA (2009). Preeclampsia: the role of angiogenic factors in its pathogenesis. Physiology..

[54] Espinoza J (2010). Angiogenic imbalances: the obstestric perspective. Am. J. Obstet. Gynecol..

[55] Cross J, Simmons DG, Watson ED (2003). Chorioallantoic morphogenesis and formation of the placenta villous tree. Annals of NY Acad. Sci..

[56] Bainbridge SA (2012). Effects of reduces Gcm1 expression on trophoblast morphology, fetoplacenta vascularity, and pregnancy outcomes in mice. Hypertension..

[57] Rennie MY, Rahman A, Whiteley KJ, Sled JG, Adamson SL (2015). Site-specific increases in utero- and fetoplacental arterial vascular resistance in eNOS-deficient mice due to impaired arterial enlargement. Biol. Reprod..

[58] Conroy AL (2013). Complement activation and the resulting placental vascular insufficiency drives fetal growth restriction associated with placental malaria. Cell Host. Microb..

[59] Krebs C (1996). Intrauterine growth restriction with absent end-diastolic flow velocity in the umbilical artery is associated with maldevelopment of the placental terminal villous tree. Am. J. Obstet. Gynecol..

[60] Todros T (1999). Umbilical Doppler waveforms and placental villous angiogenesis in pregnancies complicated by fetal growth restriction. Obstet. Gynecol..

[61] Dunk, C. & Ahmed, A. Growth Factor regulators of placental angiogenesis, in Intra uterine growth restriction, P. Baker, J. C. P. Kingdom, Eds. Springer Verlag London Ltd. Chapter 8, 149–164 (1999).

[62] Albini A (1996). The angiogenesis induced by HIV-1 tat protein is mediated by the Flk-1/KDR receptor on vascular endothelial cells. Nat. Medicine..

[63] Livak KJ, Schmittgen TD (2001). Analysis of relative gene expression data using real-time quantitative PCR and the 2(−Delta Delta C(T)) method. Methods..

[64] Kibschull M, Colaco K, Matysiak-Zablocki E, Winterhager E, Lye SJ (2014). Connexin31.1 (Gjb5) deficiency blocks trophoblast stem cell differentiation and delays placental development. Stem Cells Dev..

[65] Fong GH, Klingensmith J, Wood CR, Rossant J, Breitman ML (1996). Regulation of flt-1 expression during mouse embryogenesis suggests a role in the establishment of vascular endothelium. Dev. Dyn..

[66] Basyuk E (1999). Murine Gcm1 gene is expressed in a subset of placental trophobalst cells. Dev. Dyn..

[67] Rennie M, Whiteley KJ, Kulandavelu S, Adamson SL, Sled JG (2007). 3D visualization and quantification by microcomputed tomography of late gestational changes in the arterial and venous feto-placental vasculature of the mouse. Placenta..

[68] Whiteley KJ, Pfarrer CD, Adamson SL (2006). Vascular corrosion casting of the uteroplacental and fetoplacental vasculature in mice. Methods Mol. Med..

