# Supplementary material for: HIV antiretroviral exposure in pregnancy induces detrimental placenta vascular changes that are rescued by progesterone supplementation
Source: Sci Rep. 2018 Apr 26;8:6552. doi: 10.1038/s41598-018-24680-w (PMC5919912; doi:10.1038/s41598-018-24680-w)
Supplement: Supplementary file 1 — Supplemental figures and tables [file 41598_2018_24680_MOESM1_ESM.pdf]

HIV antiretroviral exposure in pregnancy induces detrimental placenta vascular changes that are rescued by progesterone supplementation.

Hakimeh Mohammadi[1], Eszter Papp[1], Lindsay Cahill[2], Monique Rennie[2], Nicole Banko[1], Lakmini Pinnaduwage[3], Janice Lee[1], Mark Kibschull[3], Caroline Dunk[3], John G. Sled[2,4,5], Lena Serghides[1,6,7].

[1]Toronto General Hospital Research Institute, University Health Network, 101 College Street, Toronto, Ontario, Canada, M5G 1L7.

[2]Mouse Imaging Centre, The Hospital for Sick Children, 25 Orde Street, Toronto, Ontario, Canada, M5T 3H7.

[3]Research Centre for Women's and Infants Health, Lunenfeld Tanenbaum Research Institute, Mount Sinai Hospital, 600 University Avenue, Toronto, Ontario, Canada, M5G 1X5.

[4]Department of Medical Biophysics, University of Toronto, 101 College Street, Toronto, Ontario, Canada, M5G 1L7.

[5]Translational Medicine, The Hospital for Sick Children, 686 Bay Street, Toronto, Ontario, Canada, M5G 0A4.

[6]Department of Immunology and Institute of Medical Sciences, University of Toronto, 1 King's College Circle, Toronto, Ontario, Canada, M5S 1A8.

[7]Women's College Research Institute, Women's College Hospital, 76 Grenville Street, Toronto, Ontario, Canada, M5S 1B2.

Table S1: Geometry of the feto-placental arterial tree in control and cART treated mice

	Control	cART	p-value
**Total segments (#)**	2246 (487)	5909 (1508)	**0.0006**
**Min diameter in tree (mm)**	0.0283 (0.0017)	0.0256 (0.0017)	**0.037**
**Max diameter in tree (mm)**	0.32 (0.079)	0.34 (0.063)	0.55
**Ave vessel length (mm)**	0.18 (0.024)	0.13 (0.0055)	**0.0006**
**Total vessel length (mm)**	403 (58)	768 (182)	**0.0021**

Table S2: Primer sequences

Gene	Forward	Reverse
Vegf	CAT CTT CAA GCC GTC CTG TGT	ACT CCA GGG CTT CAT CGT TAC A
Plgf	TGC TGG TCA TGA AGC TGT TC	GGA CAC AGG ACG GAC TGA AT
sFlt-1	AGG TGA GCA CTG CGG CA	ATG AGT CCT TTA ATG TTT GAC
mFlt-1	TTC ACC ATC CCA AGG CAG TC	TGC TGT GCC AAG GAG CAT AA
Flk-1	AAC GGC ACC TCT GTG AAA CT	TCC TTC CTC CCA GTC CAC AT
Ang-1	CCT CTG GTG AAT ATT GGC TTG GGA	AGC ATG TAC TGC CTC TGA CTG GTT
Ang-2	AGA GTA CTG GCT GGG CAA TGA GTT	TTC CCA GTC CTT CAG CTG GAT CTT
Gcm-1	CTG ACA AGA AGG ACA GAC TCG G	AGA GCA GCT GAA GGG CTT GT
Hprt1	AGC GTC GTG ATT AGC GAT GA	ACA CTT TTT CCA AAT CCT CGG C

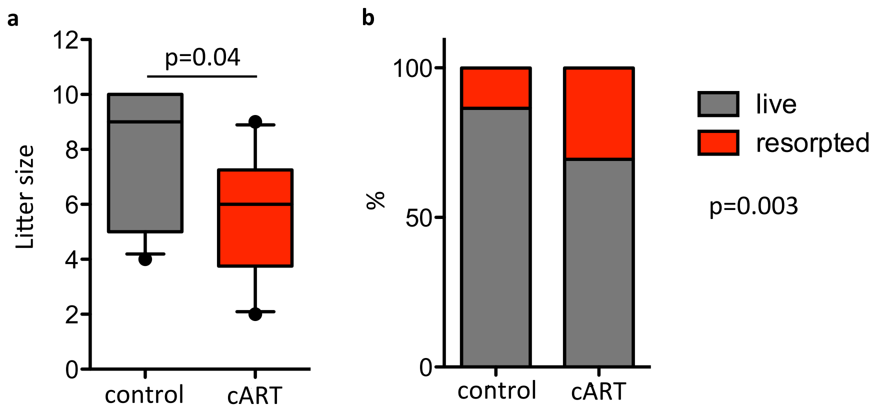

Fig. S1. Litter size is smaller and number of resorptions is greater in cART-exposed pregnant mice.

Pregnant mice were treated by gavage with cART (zidovudine/lamivudine/lopinavir/ritonavir) or water as a control starting on GD1 until sacrifice on GD15. Litter size (a) and number of resorptions (b) were assessed on GD15. For (a) data shown as box (showing median and interquartile range) and whisker (showing 10th to 90th centile). Dots signify values outside the 10th and 90th percentile. Statistical comparison by Mann Whitney U test. For (b) the grey bars indicate the percentage of live fetuses and the red bars the percentage of resorptions. Statistical analysis by Fisher's exact test.

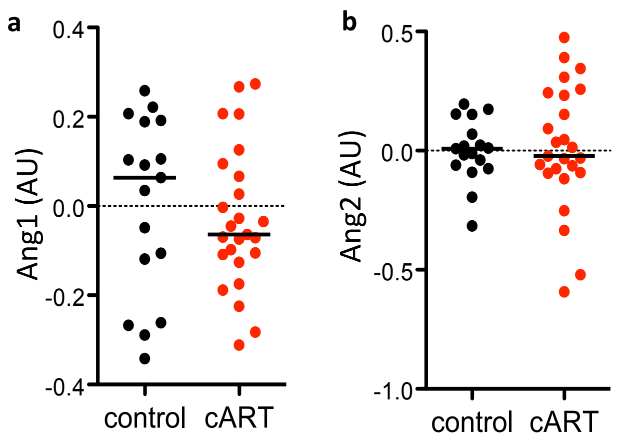

Fig. S2. Placental expression levels of angiopoietin 1 and 2 are similar between control and cART-exposed mice.

(a) Angiopoietin-1 (Ang1) and (b) angiopoieting-2 (Ang2) expression levels (log transformed) assessed by qPCR in gestational day 15 placenta of mice exposed to water as a control (black dots) or cART (red dots) starting on gestational day 1 of pregnancy. The lines indicate the median. Statistical comparison by Mann Whitney U test. AU, arbitrary units.
